# Threshold selection in gene co-expression networks using spectral graph theory techniques

**DOI:** 10.1186/1471-2105-10-S11-S4

**Published:** 2009-10-08

**Authors:** Andy D Perkins, Michael A Langston

**Affiliations:** 1 Department of Computer Science and Engineering, Mississippi State University, Mississippi State, MS, USA; 2 Department of Electrical Engineering and Computer Science, University of Tennessee, Knoxville, TN, USA

## Abstract

**Background:**

Gene co-expression networks are often constructed by computing some measure of similarity between expression levels of gene transcripts and subsequently applying a high-pass filter to remove all but the most likely biologically-significant relationships. The selection of this expression threshold necessarily has a significant effect on any conclusions derived from the resulting network. Many approaches have been taken to choose an appropriate threshold, among them computing levels of statistical significance, accepting only the top one percent of relationships, and selecting an arbitrary expression cutoff.

**Results:**

We apply spectral graph theory methods to develop a systematic method for threshold selection. Eigenvalues and eigenvectors are computed for a transformation of the adjacency matrix of the network constructed at various threshold values. From these, we use a basic spectral clustering method to examine the set of gene-gene relationships and select a threshold dependent upon the community structure of the data. This approach is applied to two well-studied microarray data sets from *Homo sapiens *and *Saccharomyces cerevisiae*.

**Conclusion:**

This method presents a systematic, data-based alternative to using more artificial cutoff values and results in a more conservative approach to threshold selection than some other popular techniques such as retaining only statistically-significant relationships or setting a cutoff to include a percentage of the highest correlations.

## Background

The construction of gene co-expression networks is often a necessary step in a bioinformatic analysis of microarray gene expression data. Studies have shown that genes showing a similar pattern of expression, those sharing edges in a co-expression network, tend to have similar function [1]. This principle, often referred to as "guilt-by-association" is the idea that motivates many microarray studies. With new high-throughput sequencing technologies currently being used for digital gene expression applications, gene co-expression networks promise to continue to find wide utility in genome-wide association studies and other computational analyses.

These networks are constructed by computing some similarity value between gene transcripts based upon their expression values over a set of samples. Nodes in the network represent transcripts while edges are weighted by these similarity values. A threshold is often applied to the resulting networks to retain only the most biologically significant relationships. This threshold application step is a major juncture in which errors can be introduced in the form of both false negatives and false positives. By setting this threshold too high, important relationships can be lost. Likewise, we must be sure to remove connections that do not represent "real" relationships. This task is difficult since the range of thresholds representing real biological relationships that also avoid over-filtering can be narrow.

Some of the many methods that have been applied to the threshold selection problem in various types of networks are using an arbitrary threshold [2], retaining only the top *x *percent of the strongest relationships [3], permutation testing [4], and filtering based upon control spot correlations [5] or the statistical significance of the relationships [5-7]. The method presented here makes use of initial spectral graph theory-based clusterings to help identify an appropriate threshold. Combinatorial methods such as those described in [5] will be used to analyze the final gene co-expression network, and such methods often require significant computational resources. We can justify the expense of this initial clustering by the computational resources saved by picking a suitable threshold in advance, especially one that removes most non-biologically-relevant relationships, which will significantly decrease computational requirements. We know that spectral graph theory methods can give us important information on the structure of a graph, such as the number of connected components, information about random walks in the graph, and a bound on the graph diameter [8]. Various spectral methods have also been employed to identify clusters of related vertices [9-12]. It is these spectral clustering methods that we believe can contribute toward selecting a biologically-relevant threshold in co-expression networks. A more detailed initial analysis is presented in [13].

## Results and discussion

### Spectral properties and algebraic connectivity

We introduce a method for threshold selection based upon the spectrum of the graph at varying thresholds. That is, the eigenvalues and eigenvectors of a transform of the graph's adjacency matrix. We applied this method to yeast cell cycle data [14] and human expression values collected over many different tissue types [15]. It has been shown that the number of connected components of a network can be identified using the spectrum of the network [8]. Ding et al. observed that "nearly-disconnected" portions can also be identified by examination of the eigenvector associated with the smallest nonzero eigenvalue of the network, often called the Fiedler vector [10]. The ability to find the nearly-disconnected pieces allows us to identify those nodes sharing a well-connected, or dense, cluster.

**Figure 1:**
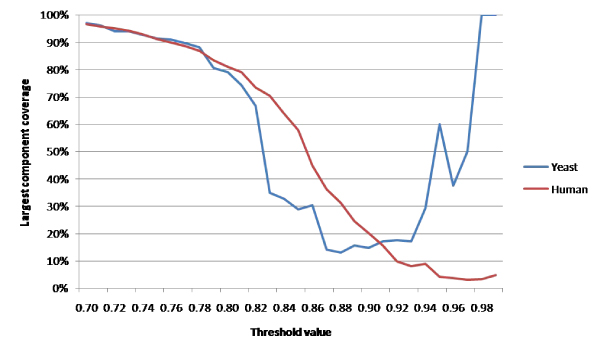
**Connected components**. The percentage of network nodes contained within the largest connected component for both yeast and human data sets.

For this study, we analyzed the spectrum of the largest connected component in networks constructed at increasingly stringent thresholds. Figure 1, which illustrates the number of vertices belonging to the largest connected component, shows that the largest component often contains a majority of the network nodes. The exception occurs at high thresholds where the network becomes very sparse. It can be shown that the multiplicity of the zero eigenvalue is equal to the number of connected components in the graph.

**Figure 2:**
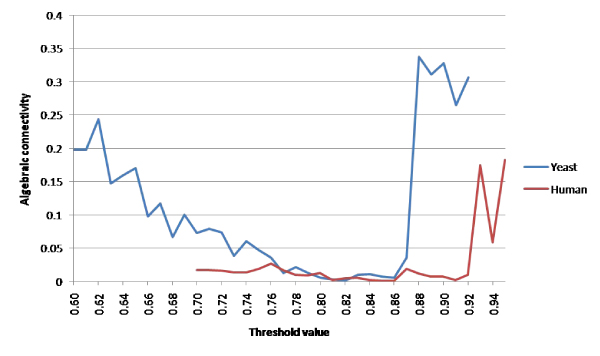
**Algebraic connectivity**. Algebraic connectivity measured at various thresholds for co-expression networks on both yeast and human data sets. Very high connectivity values falling at the extreme upper thresholds were omitted to keep the scale of the chart from overwhelming the value of other observations.

Therefore, when analyzing only the spectrum of the largest component, the smallest eigenvalue will be equal to zero while the remaining eigenvalues will be nonzero. We will use the common notation of calling this smallest nonzero eigenvalue the algebraic connectivity of the component and refer to it as *λ*_1_. Figure 2 shows the algebraic connectivity for the two data sets studied. Lower connectivity values indicate the presence of nearly-disconnected components [10]. *λ*_1 _reaches a minimum in yeast at *t *= 0.82, though it remains at a relatively low level (less than 0.05) from *t *= 0.75 to *t *= 0.87. For human, the connectivity values are much more variable, though a minimum is obtained at 0.85.

### Spectral clustering

Many spectral clustering methods exist, with possibly the simplest being a spectral bipartitioning of the network such as that described in [16]. In that case, the eigenvector associated with *λ*_1_, which we will refer to as **v**_1_, is sorted and nodes are partitioned into two groups based upon the magnitude of their associated eigenvector value. In [10], the authors showed that sorting the eigenvector associated with *λ*_1 _in ascending order often produces a step function-like plot. They also showed that the steps in such a plot delineate transitions from one nearly-disconnected component to another. Since each eigenvector value is associated with a node in the network, individual nodes can be assigned to a cluster based upon the steps in the eigenvector values. This method allows a finer partitioning than the spectral bipartitioning methods and precludes the need for recursive application of the partitioning method. This particular spectral clustering method is particularly amenable to the threshold selection problem due to its ability to identify clusters of various sizes and because it is not necessary to specify the number of partitions desired.

**Figure 3:**
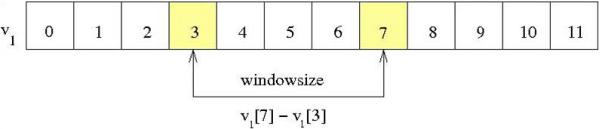
**Sliding window**. An example of a sliding window comparison to detect transitions between well-connected components.

A sliding window method, illustrated in Figure 3, was used to identify transitions from one cluster to another. Since these transitions are often not immediate, but occur over the span of several eigenvector values, a simple comparison of adjacent positions is not sufficient. Therefore, we compute the difference of eigenvector values some constant distance apart. Here we used a window size of five positions, which was observed to correctly identify most steps in the eigenvector plot.

**Figure 4:**
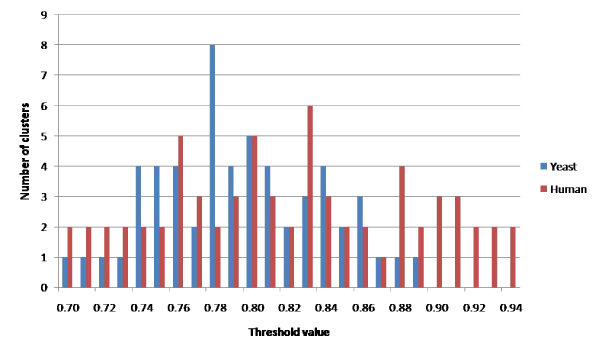
**Number of clusters**. The number of clusters identified by the spectral method in yeast and human co-expression networks at various thresholds.

Employing the principle of guilt-by-association, we know that weaker relationships should connect functionally dissimilar portions of the network. Therefore as the threshold is increased, these portions will become less connected to one another, resulting in a likely increase in "nearly-disconnected" components. We select the threshold value that maximizes the number of these components and thus minimizes the number of edges connecting these pieces. Figure 4 shows the number of clusters identified at various thresholds for both data sets. Based upon the number of clusters, the spectral graph theory-based method identified potential threshold values of 0.78 in the co-expression network on yeast data and 0.83 in the human network.

We can see in the previous figure that the number of clusters identified by the spectral method subsequently decreases as we proceed past the selected threshold. This is likely due to a decreasing network size overall, as well as individual clusters falling below the minimum cluster size. Similarly, the algebraic connectivity shown in Figure 2 shows an associated increase at the upper end of the threshold range due to the very small size of the largest component at these thresholds. For example, at the *t *= 0.98 threshold in yeast data (not shown), the network consists of only two nodes, with a single edge connecting them for a 100% edge density.

**Figure 5:**
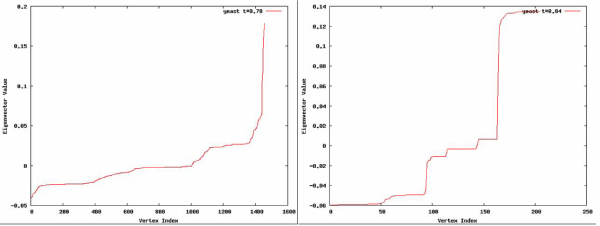
**Sorted eigenvectors**. Eigenvector values associated with the smallest nonzero eigenvalue in yeast co-expression networks at thresholds of (a) 0.78 and (b) 0.84. For each vertex, the associated eigenvector value is plotted.

Figure 5 shows the step-like structures found for two thresholds in yeast data. At the *t *= 0.78 threshold identified by the spectral method, as discussed above, the steps are not as clearly delineated as at the *t *= 0.84 threshold, also shown. While the step functions are more defined at the higher threshold, the number of nodes remaining in the network has greatly decreased and the remaining clusters have become too small to surpass our minimum cluster size requirement.

### Combinatorial analysis

Paracliques [17] were computed for co-expression networks generated at the selected thresholds for both the human and yeast data sets. The Paraclique algorithm, based upon solving the -complete clique problem [18], is often more appropriate for microarray data than using the basic clique method. Due to the noise inherent in such data, a small number of edge weights can drop just below the network threshold. Paraclique corrects such a situation by allowing vertices to be added to the paraclique if they are adjacent to at least *g *of the original clique members. For most of our analyses (except the comparison with known clusters of co-expressed yeast genes described below), we set *g *= 1. The Paraclique algorithm performs this adjustment while still retaining the benefits of clique such as being an unsupervised method, identifying only the densest subgraphs, and possessing a natural resistance to false positives.

In the yeast co-expression network at the *t *= 0.78 threshold chosen by the spectral method, 93 paracliques were found with the largest containing 21 gene transcripts. At the *t *= 0.55 threshold identified by choosing the top one percent of correlations, we found 636 paracliques with a maximum size of 93. The human network produced many more and larger paracliques, with 497 paracliques and the largest one containing 78 transcripts at the more conservative threshold of 0.83. The human network constructed over all tissues and replicates at the lower threshold of 0.65 contained 2, 843, 536 edges, and the Paraclique run extended for almost 2.9 hours on an Intel Pentium 4 EM64T 3.4 GHz processor. This graph contained 1283 paracliques, with the largest having 324 members.

### Comparison with other results

#### Traditional methods

We examined the difference between the networks generated at thresholds selected by the spectral method, retaining only the strongest one percent of relationships, and filtering by statistical significance at the *p <*0.05 and *p <*0.01 levels. The statistical significance results assume all data points are present for every pair of transcripts, which may not be the case. Table 1 shows results from each one of these methods, with the "Adj. *p <*0.05" and "Adj. *p <*0.01" columns containing significance values after adjustment for multiple tests. For both data sets, the eigenvector-based method selected a higher threshold than the other methods. While this shows that the spectral method excludes relationships that would otherwise be considered statistically significant, available computational resources and tractability of the problems involved often indicate the need to reduce the network size. For example, the network from human data at a threshold of 0.22, which would correspond to *p <*0.05 if all replicate tissues were averaged, has 106, 629,395 edges on 22, 283 vertices. Also, while correlations at this low magnitude would be categorized as significant, they are still rather weak and should be excluded to further reduce the false positive rate.

**Table 1:** Threshold values. Threshold values computed by various methods for yeast and human co-expression networks.

Threshold values						
	Spectral	*p <*0.05	*p <*0.01	Adj. *p <*0.05	Adj. *p <*0.01	1%
Yeast	0.78	0.22	0.28	0.46	0.49	0.55
Human	0.83	0.16	0.20	0.36	0.38	0.65

Table 2 lists the number of vertices and edges contained in both the yeast and human graphs at the threshold generated by the statistical significance method at both the *p <*0.05 and *p <*0.01 levels adjusted for multiple tests, as well as the spectral approach and the method of retaining the top one percent of correlations. Since the set of edges at a higher threshold is a subset of the edges at each of the lower thresholds, it is easy to see the number of relationships filtered out by each subsequent increase in threshold value. While the yeast data set is small and does not pose a significant challenge to the computation resources available, we can see that graphs constructed on the human data set become very large as the threshold is decreased. We will see that the problem becomes difficult to solve on a single processor even at the threshold selected by the relatively conservative method of retaining the top one percent of relationships.

**Table 2:** Vertex and edge counts. The numbers of vertices and edges in graphs constructed at thresholds identified by various methods.

Vertex and edge counts								
	Spectral		Adj. *p <*0.05		Adj. *p <*0.01		1%	

	Vertices	Edges	Vertices	Edges	Vertices	Edges	Vertices	Edges

Yeast	1652	4746	6177	665, 859	6174	463, 000	6108	212, 127
Human	6163	66, 126	22, 283	50, 202, 163	22, 283	44, 057, 599	17, 757	2, 843, 536

To correct for multiple tests, we apply the method described in [5]. For example, the *α *= 0.05 significance level was divided by the number of transcripts on the array. The normal quantile function was used, followed by an inverse Fisher's *z' *transformation to determine the associated correlation value. This adjustment for multiple comparisons increases the standard p-value slightly, though the significance level threshold is still very low. Such large sample sizes (*n *= 82, yeast; *n *= 158, human) tend to translate into low correlation values required for significance, even with adjustment.

#### Previous studies

Other spectral techniques have also previously found utility in addressing in the network threshold problem. Nearest neighbor eigenvalue spacing was used in [19] to employ random matrix theory methods for threshold selection. Here, the authors analyzed the eigenvalues of the network by examining the distribution of spacings between successive eigenvalues and determined the point at which this spacing distribution transitioned from Poisson to Gaussian Orthogonal Ensemble (GOE). [19] also studied the yeast dataset described in [14] and found that the transition began at *t *= 0.62 and was complete by *t *= 0.77. For this yeast data set, the identification of the *t *= 0.77 point corresponds approximately to our result of *t *= 0.78.

Much information is provided about the co-expression of yeast genes over the cell cycle in [14]. We compared Paraclique results with seven of the clusters of genes identified by the authors that had similar expression levels over the cell cycle. Paracliques were enumerated at the 0.78 threshold identified by the spectral method, with additional vertices being added to the paraclique if they were adjacent to at least three of the original clique members. A preliminary examination of the Paraclique results uncovered several paracliques containing portions of these clusters of genes known to be co-expressed over the cell cycle, according to [14]. A summary of the results is given in Table 3. Note that all of these comparisons were performed on an abbreviated set of cluster genes present in the heatmaps in the [14] manuscript. Single paracliques were found to contain genes from both the histone and CLB2 clusters, with 8/9 histone genes accounted for and 10/36 CLB2 genes. Paracliques also contained CLN2 and Y' cluster genes (25/58 and 19/27, respectively) though genes from each of these two clusters spanned two distinct paracliques. None of the genes from the MET, MCM, or SIC1 clusters were found. The conservative threshold value of 0.78 along with the stringency of the paraclique algorithm likely precluded the appearance of the MET, MCM, and SIC1 cluster genes. The method here was able to identify several known cell cycle-regulated genes, particularly those [14] identified as forming the "tightest cluster", the histones. A more comprehensive set of comparisons utilizing all of the co-expressed genes identified in [14] as well as other sources of known co-expressed cycle cycle genes will be necessary to draw any significant conclusions. In [20], the author examined the spectral threshold selection method along with other approaches in a bootstrap analysis on three yeast data sets. The study found that the spectral threshold method produced thresholds of 0.93, 0.97, and 0.89 on yeast anoxia and reoxygenation [21] and yeast alpha-factor arrest [14] data sets, respectively. Networks constructed at these thresholds contained maximum cliques of sizes 73, 17, and 15.

**Table 3:** Comparison with known co-expressed yeast genes

Cluster and paraclique overlap			
Cluster (from [14])	Number of genes in cluster (abbreviated-from heat map figures)	Number of paracliques with cluster overlap	Total paraclique overlap

CLN2	58	2	25
Y'	27	2	19
Histone	9	1	8
MET	20	0	0
CLB2	36	1	10
MCM	38	0	0
SIC1	27	0	0

A list of clusters of genes found to be co-expressed over the yeast cell cycle in [14], along with the number of paracliques found to contain those genes in this study and the total number of cluster genes found in all paracliques. Comparisons were performed on an abbreviated set of cluster genes specified in the heatmap figures from [14].

### Functional comparisons

Due to the nature of the data set analyzed, genes existing in dense regions of the human co-expression network will be those that show the same pattern of expression over many tissue types, though not necessarily over- or under-expressed in a single tissue type. Similarity in many samples is likely required to drive correlations to a significant level. Similarly, genes identified to be in paracliques in the yeast data set are those that vary together throughout the cell cycle. We used the GO Slim Mapper at the Saccharomyces Genome Database (SGD) [22] and Ingenuity Pathways Analysis (Ingenuity Systems, http://www.ingenuity.com) to analyze some resulting paracliques in yeast and human, respectively.

In the yeast networks, we examined the biological process gene ontology category for the three largest paracliques and identified categories for which more than three genes appeared. At the *t *= 0.78 threshold, these paracliques were of size 21, 17, and 15. For the largest paraclique, nine of the 21 genes had unknown molecular function; 7, hydrolase activity category; 6, helicase activity; 3, RNA binding. The second paraclique showed categories of DNA binding, enzyme regulator activity, and hydrolase activity. All genes in the third appeared in the structural molecule activity category, and five in RNA binding. The three largest paracliques at the lower threshold of *t *= 0.55 identified by the top one percent of correlations method were of size 93, 53, and 37, respectively. Many more of these genes were found to have unknown molecular function (40, 13, and 17). The first also contained genes related to hydrolase activity, RNA binding, helicase activity, transferase activity, and nucleotidyltransferase activity. Those with more than three members in the second paraclique were transferase activity, DNA binding, hydrolase, enzyme regulator activity, protein binding, and protein kinase activity. Protein binding, hydrolase, and RNA binding were identified in the third paraclique.

For human paraclique results, we also examined the three largest paracliques at the thresholds identified by the spectral method and the "top 1%" method. At the *t *= 0.83 threshold, the first paraclique matched five networks containing more than three of the paraclique members. These included networks related to cellular organization, gene expression, genetic disorder, drug metabolism, and cell signaling, for example. The second paraclique matched only three networks, all related to protein synthesis. Similarly, the third network aligned with only two networks with a match of more than one gene. Both of these were related to reproductive systems development and disease, respectively, among other functions. The *t *= 0.65 threshold produced a maximum paraclique size of 324 which matched 14 networks with more than three genes in common, with the most enriched being related to post-transcriptional modification. The second largest paraclique matched 13 networks ranging from cellular assembly and organization, genetic disorder, to inflammatory disease, and many others. Similarly, the third paraclique matched nine networks, mostly having some relation to cancer, though some were annotated with cellular development, post-translational modification, and developmental/genetic disorder, for example.

For yeast results, while paracliques computed at the higher threshold of *t *= 0.78 are understandably smaller, fewer genes are unidentified based upon their biological process. In one case, all of the genes in a paraclique fell into the same category. In paracliques on networks constructed at both the high and low thresholds, genes belonged to a wide variety of biological processes, and largely the same categories appeared within the three largest paracliques in both groups. IPA results on the three largest human paracliques shows that lower thresholds result in a larger number of networks matching the paraclique transcripts. These networks seem to be annotated with a larger range of functions compared to the relatively few networks identified at the higher thresholds. In this sense, it is possible that the higher threshold values produce paracliques that are more specific to a particular network or function, allowing us to examine the results at a finer granularity. Of course, analyzing only the three largest paracliques does not give enough information to draw definitive conclusions, and it is likely that some of the actual biological networks involved or genes belonging to these networks will have been lost by using a more stringent threshold.

### Threshold effect on co-expression networks

With respect to an individual paraclique, an increase in correlation threshold can have have at least two effects, and possibly both of these: a decrease in the number of genes contained within a paraclique, or the splitting of a paraclique into two or more disjoint paracliques. These new disjoint pieces may contain additional genes that were not present in the original paraclique due to the smaller number of genes with which a new gene would have to share a connection. Both of these cases are possibly desirable when a large paraclique encompasses genes participating in a variety of biological functions. If that large paraclique is split into multiple disjoint pieces of highly connected genes, or genes connected to the paraclique at a lower correlation level are excluded, only the core set of genes putatively involved in a more focused set of biological functions or pathways remain.

We decided to identify occurrences of each of these cases in the human data set due to the availability of the rich annotation information for human results available within IPA. Using the combinatorial methods described above can become intractable at the very low threshold values corresponding to large numbers of vertices and edges identified by the statistical significance methods. Therefore, we performed a pairwise comparison between paracliques computed at the two highest threshold values selected by all of the methods studied. The degree of overlap between each paracliques in the graph constructed by choosing the highest one percent of correlations (0.65) and each of those identified at the higher spectral threshold (0.83) was found. This allowed us to determine which paracliques at the higher threshold possibly correspond to those in the graph at the lower threshold. Note that due to the nature of the Paraclique algorithm, there is not necessarily a one-to-one correspondence between every paraclique in the first set with one or more paracliques in the second set.

Table 4 illustrates the case in which the number of transcripts in a paraclique was decreased when moving from a lower to a higher threshold value. A paraclique containing 24 transcripts computed at the higher threshold was found to be completely contained within a paraclique of size 47 at the lower threshold. While the paraclique at the higher threshold was much smaller, both sets of transcripts mapped to approximately the same set of IPA focus molecules, and therefore matched similar network functions. However, due to the reduced input size, the associated p-values for enrichment in many of the biological functions were reduced (not shown). After mapping transcript IDs to gene symbols, it appeared that the increase in threshold excluded mostly a few uncharacterized genes from the paraclique.

**Table 4:** IPA networks from two paracliques

IPA networks			
Paraclique	Threshold	Unique genes	Network functions

1	0.65	21	Hematological System Development and Function, Humoral Immune Response, Tissue Morphology Cellular Movement, Embryonic Development, Hair and Skin Development and Function
2	0.83	13	Hematological System Development and Function, Humoral Immune Response, Tissue Morphology Cellular Movement, Embryonic Development, Hair and Skin Development and Function

Paraclique 1 was extracted from a graph constructed at the 0.65 threshold while the smaller paraclique 2 is from a graph at the 0.83 threshold level. IPA indicates that genes from both of these gene sets match a similar set of network functions. In both cases, the set of focus molecules identified by IPA consisted of IGHD, IGHG1, IGHM, IGKC, and IGL for the first network and IGKV1–5 for the second.

**Figure 6:**
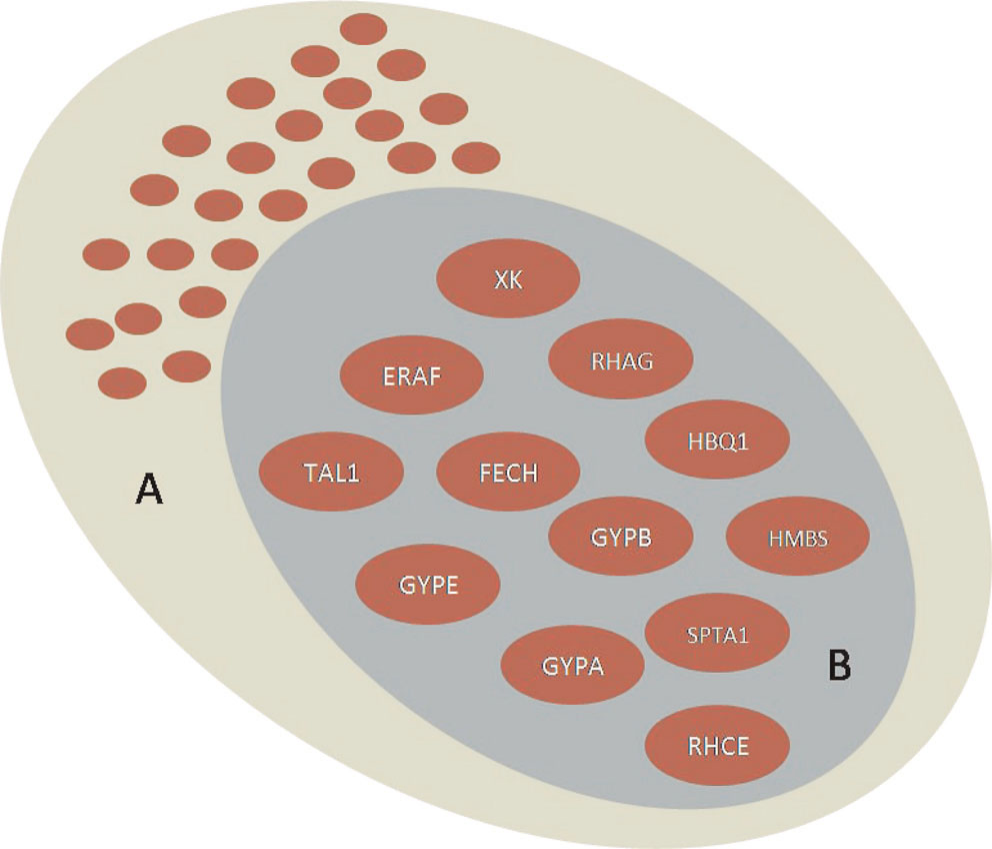
**Paraclique containment**. The large green paraclique of 51 transcripts, A, was computed at the 0.65 threshold. Paraclique B, identified from the graph at a 0.83 threshold, contained 17 transcripts. After converting to gene symbols, A and B had an intersection of size 12 genes. The remaining genes from paraclique A may be present in other, smaller paracliques found at the 0.83 level. IPA showed that paraclique A matched several possible networks, while the smaller paraclique B matched only a single network associated with cellular development, hematological disease, and cell morphology.

There are cases in which annotations for a large paraclique can be convoluted and hard to interpret. Figure 6 shows a paraclique containing 51 transcripts (representing 36 genes) at the 0.65 threshold. This paraclique shares all of the 17 transcripts (12 genes) present in a paraclique at the higher 0.83 threshold. While the larger set of genes matched five top-scoring network functions in IPA, the smaller set matched only a single network related to cellular development, hematological disease, and cell morphology which was also the top-scoring network at the 0.65 level. The ability to analyze these gene sets at finer levels of granularity greatly increases the confidence with which we can interpret the results.

**Figure 7:**
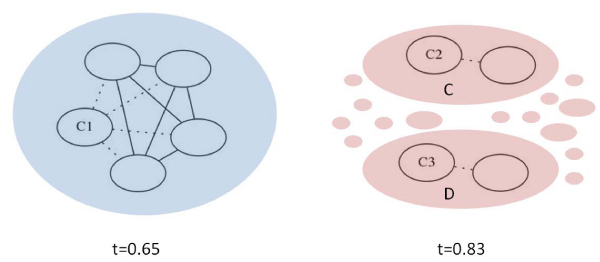
**Paraclique decomposition**. The large paraclique on the left, identified at the 0.65 one percent threshold, contained 154 gene transcripts. 150 of these were contained in the core clique *C*1. Sixteen paracliques were found at the 0.83 spectral threshold with an intersection with this large paraclique of at least one transcript. The largest of these, labeled C and D, were of size 54 and 42, respectively. All 54 genes in paraclique C were contained in the paraclique on the left, while the intersection with D was of size 35. Edges may exist between members of the different paracliques, but are not shown for readability. Dotted lines indicate that not all connections are present between a gene and the core clique, since the paraclique requires all but one possible connection between a gene and the core maximal clique.

The large paraclique at the left in Figure 7 was identified at the 0.65 threshold and was found to be "split" into two main components at the 0.83 threshold. While both of these components contain mostly ribosomal protein transcripts, 1170 edges were lost between the two groups by raising the threshold. Since the Paraclique method at the selected stringency requires that all but one connection exist between all paraclique members, the larger paraclique was decomposed into the two main components with a high proportion of genes overlapping with the original paraclique (54/54 and 35/42, respectively) on the right of the figure as well as several smaller pieces with an overlap of between 1 and 13 transcripts. The average correlation of the remaining edges within the two smaller paracliques was around 0.90.

## Conclusion

We have presented a systematic threshold selection method that makes use of spectral graph theory techniques. We have shown that in the selected data sets this method results in a more conservative approach to threshold selection than both the test of statistical significance at *p <*0.01 and including only the highest-weighted 1% of edges, in terms of the number of relationships retained for further analysis. We believe that the primary strength of the spectral graph theory-based method presented here is that it is a systematic method for threshold selection. Both the statistical significance method and the percentage cutoff method can be adjusted to produce graphs that prove to be tractable in a combinatorial analysis and contain fewer false positives, but the need for an arbitrary cutoff value is still present in these methods. The spectral approach attempts to move beyond the need for employing these arbitrary thresholds and computes a cutoff value based upon the underlying community structure of the data rather than merely sample size or the relative distribution of correlation values. We have also shown that for the yeast cell cycle data studied, this method produces results in agreement with a previous study making use of methods from random matrix theory. Functional comparisons between networks constructed at the threshold selected by the spectral method and the method of choosing the top one percent of correlations show that the networks built at the lower threshold are often time consuming to analyze and in the yeast data set, many of the paraclique members fall into the unknown biological process category while other genes span several other GO categories. At the higher threshold, fewer of these genes fail to be categorized based upon the gene ontology. For human data, fewer networks were identified as being enriched in the paracliques, making interpretation of the results easier.

Future work may include adapting more advanced spectral clustering methods such as the *k*-way partitioning methods described in [9,11,12] for use in threshold selection. We also plan to investigate the use of the metric of modularity [23], which serves as a quantitative measure of the proportion of intra-cluster edges, as a guide for determining an optimal threshold. Both of these features can be incorporated into a future graphical user interface-based software package that can be applied to general microarray data sets to perform a spectral analysis for determining an appropriate threshold.

## Methods

### Microarray data sets

We studied the publicly-available *Homo sapiens *and *Saccharomyces cerevisiae *microarray data sets described in [15] and [14], respectively. The former contains expression values from a panel of seventy-nine different tissue types in human measured on Affymetrix HG_U133A gene expression microarrays at the Genomics Institute of the Novartis Research Foundation (GNF). Data was downloaded from the NCBI Gene Expression Omnibus website http://www.ncbi.nlm.nih.gov/geo/ as raw CEL files and subsequently preprocessed and normalized using the R statistical software package version 2.6.1 [24] and the justRMA() function of the affy version 1.12.2 [25] Bioconductor [26] package. The latter contains expression from baker's yeast samples collected over a time period to measure changes during the cell cycle and was downloaded from the author's webpage in tab delimited format.

### Network construction and representation

We constructed a gene co-expression network at increasingly stringent thresholds by beginning with a complete graph with vertices representing gene transcripts. The Pearson product-moment correlation coefficient was computed between each pair of transcripts with at least 10 data observations in common and used to weight the appropriate network edge. A high-pass filter was subsequently applied to the absolute value of each edge weight, removing those edges with an absolute weight less than some threshold *t*. As *t *proceeded from 0.70 to 0.95, a co-expression network was constructed at each threshold value. Traditional non-spectral methods were used to identify connected components within the network and extract the largest for spectral analysis. The resulting unweighted graph *G *= (*V*, *E*) can be represented by its adjacency matrix, given by

We define a transform of the adjacency matrix, the Laplacian of the graph *G*, as in [8] by

where *deg*(*i*) denotes the degree of vertex *i*. The benefit of the Laplacian matrix is that both adjacency and degree information is readily available.

### Eigenvalue and eigenvector computation

We aim to solve the eigenvalue problem on the Laplacian matrix defined above. Using notation similar to [10], this involves solving the system of equations

resulting in the eigenvalues

and associated eigenvectors

where *n *is the number of nodes in the component being analyzed.

The linear algebra software package MATLAB version R2008b (The Mathworks, Inc., http://www.mathworks.com) was used to compute approximations to selected eigenvalues and eigenvectors of the filtered correlation network. Using the sparse matrix operations native to MATLAB and the eigs() function, the two smallest eigenvalues and their associated eigenvectors were computed. The eigenvector associated with the second-smallest eigenvalue *λ*_1 _was extracted and sorted in increasing order.

### Cluster detection

The detection of "gaps" in the ordered set of eigenvector values was performed using a sliding window technique. The sliding window compares two eigenvector values windowsize positions apart, where windowsize was chosen to be five for this study. When these two values are significantly different, then the beginning of a new cluster is indicated. In this case, we define a significant difference to be greater than *m *+ , where *m *is the median of all differences in positions windowsize apart and *s *is the standard deviation of this set of values. To prevent the many small partitions that often occur at extremely high thresholds from overwhelming the results, identified partitions less than some minimum size, in this case 10 nodes, were discarded.

### Paraclique extraction

The graph theoretical algorithm Paraclique, developed by Michael A. Langston's team at the University of Tennessee and described in [17], was employed to extract dense sets of genes from resulting co-expression networks. Paraclique begins by finding a clique, or completely connected subgraph, of maximum size in the network. The maximum clique is augmented with genes connected to all but *g *of the clique members, with *g *= 1 in this case. This dense subgraph is removed from the network and the process repeats until no new paracliques larger than some minimum size can be found. For comparisons with known yeast co-expression networks, we set *g *= 3, which was found to incorporate more of the known co-expressed genes without significantly increasing the number of other genes present. We required the base maximum clique size to consist of at least five members for the comparison with previous yeast co-expression studies and three for all other human and yeast results.

### Functional comparisons

Functional comparisons were performed using the Saccharomyces Genome Database GO Slim Viewer http://www.yeastgenome.org and Ingenuity Pathways Analysis software (Ingenuity Systems, http://www.ingenuity.com) for yeast and human networks, respectively.

## Competing interests

The authors declare that they have no competing interests.

## Authors' contributions

ADP and MAL designed the project. ADP performed the experiments, analyzed results, and drafted the paper. MAL assisted with revisions. Both authors reviewed and approved the final manuscript.
